# Depression risk associated with the use of 5α-reductase inhibitors versus α-blockers: A retrospective cohort study in South Korea

**DOI:** 10.1371/journal.pone.0265169

**Published:** 2022-03-16

**Authors:** Bora Yeon, Ah Young Suh, Eunmi Choi, Bonggi Kim, Eunsun Noh, Soo Youn Chung, Soon Young Han

**Affiliations:** 1 Department of Drug Safety Information, Korea Institute of Drug Safety and Risk Management, Gyeonggi-do, Republic of Korea; 2 Department of Relief of Adverse Drug Reaction, Korea Institute of Drug Safety and Risk Management, Gyeonggi-do, Republic of Korea; University Medical Center Utrecht, NETHERLANDS

## Abstract

**Background:**

One of the most prescribed treatments for benign prostatic hyperplasia (BPH) is 5α-reductase inhibitors (5ARI). Europe experienced recent safety issues involving 5ARI and depression symptoms, with similar findings being seen in Western countries. The South Korea has updated the drug label in accordance with European recommendations, but the relevant evidence was insufficient. This study compared the use of 5ARI versus α-blocker (AB) as a treatment for BPH and related risks of depression to provide evidence based on the Korean population.

**Methods:**

This was a retrospective cohort study using South Korea’s Health Insurance Review & Assessment Service claim data from 2011 to 2017. New patients diagnosed in men with BPH and taking medications that contained either 5ARI or AB between July 1, 2013, and June 30, 2015, were included (n = 1,461 5ARI; n = 18,650 AB). The primary outcome was depression defined per the 10th revision of the International Statistical Classification of Diseases and Related Health Problems (ICD-10: F32-34, F38, F412, F432). Logistic regression was used to implement 1:1 propensity score (PS) matching of patients taking 5ARI to those taking AB to adjust for confounding. Cox proportional hazard models were used to compare the risk of depression associated with 5ARI versus AB.

**Results:**

Balance in baseline characteristics between the treatment groups were achieved within PS matched pairs (1,461 pairs). Compared to the AB medication group, the 5ARI group had lower depression (HR: 0.69, 95% CI: [0.51–0.92]). However, we could not find a clinically relevant, statistical difference after PS matching (HR: 0.91, 95% CI: [0.61–1.36]).

**Conclusions:**

The risk of depression associated with 5ARI was not meaningfully different from AB in Korea, which suggests that medical officials should provide the most appropriate medication for BPH patients by considering both treatment benefits and depression risk.

## Introduction

BPH is a genitourinary disorder in which the size of the prostate around the urethra increases, and the urethra narrows or becomes clogged, causing dysuria. BPH is a common disorder among people over 50 years old and accounts for 25% of the total urologic diseases [1]. Due to a rapidly aging population, BPH (ICD-10: N40.0, N40.1, N40.2, N40.3, N40.8) prevalence is continually increasing estimated to afflict 50% of males in their fifties, 60% in their sixties, and up to 80% for males over eighty years old (according to a previous study using Korean insurance claims data from 2006 to 2011) [2]. Common manifestations of BPH include lower urinary tract symptoms (LUTS)—such as urinary frequency, incomplete emptying, and nocturia [3]—that deteriorate the quality of life for patients with BPH [4, 5].

One of the most commonly used medications to treat benign prostatic hyperplasia (BPH) is 5α-reductase inhibitors (5ARI). Long-term 5ARI therapy is recommended for patients with moderate to severe lower urinary tract symptoms with an enlarged prostate or risk of BPH progression. Alpha-adrenergic blockers (AB) are another first-line treatment for BPH, and anticholinergic drugs are available for patients who experience bladder storage symptoms, including urgency, nocturia, and urinary frequency [6]. The indications and usual dosage of these drugs in Korea are generally similar to those in Western countries [7–9].

Production of dihydrotestosterone, which plays an essential role in the growth and development of the prostate, is inhibited by 5ARI [10]. Since neuroactive steroids such as dihydrotestosterone (DHT) and allopregnanolone are correlated with mood symptoms, including depression, 5ARI may lead to the development of mood swings, including depression [11].

The European Medicines Agency (EMA) recommended to include a warning about depression in the product information of finasteride in 2017 [12]. In addition, there have been safety issues related to depression in other countries, including the United States and Canada [13–17]. Following that recommendation, South Korea’s Ministry of Food and Drug Safety (MFDS) has updated the drug label of finasteride among 5ARI ingredients, adding information about mood alterations, including depression and suicidal thoughts in the warning section [18].

The prior study [17–20] reported the occurrence of depression after 5ARI use, and another study [21, 22] showed that there was no association between 5ARI use and depression. Despite that, some studies have been conducted assessing the relationship between 5ARI use and the occurrence of depression, suicide, and self-injury, there is only one population-based study in Korea on BPH patients who visited the urology department at a single university hospital [23]. In South Korea, drug labels have been updated due to foreign issues, but studies based on the Korean population are insufficient. Therefore, this study focused on assessing the relationship between 5ARI use and depression compared to AB in the South Korean population.

## Methods

### Design and setting

We conducted a retrospective cohort study using nationwide claims data from the Health Insurance Review and Assessment Service (HIRA) in South Korea. The claims data contains general patient information, healthcare services provided to inpatients, diagnoses (per the 10th revision of the International Statistical Classification of Diseases and Related Health Problems [ICD-10]), and outpatient prescriptions for each individual. The insurance claims database, including patient information, was anonymized and a remote access environment setup for the analysis. Also, it is a secondary data source that is collected and used for only research or public purposes, such as the prevention of national diseases and establishment of health care policies, and does not require the consent of the participants. The researchers used the de-identified secondary data in three stages (anonymization variables, grouping, masking) provided by HIRA. Therefore, the institutional review board of the Korea Institute of Drug Safety & Risk Management (KIDS) exempted the need for consent through expedited deliberation and approved this study. Patient informed consent was not required because the anonymized data did not contain any personal information.

### Data source

The health insurance claims data (generated when healthcare providers submit for reimbursement of healthcare services) was used for this study. The data includes about 98% of all the national population due to the universal coverage system of Korea (97% health insurance and 3% medical aid). More than 99% of claim data is collected in an electronic data interchange system and contained by about 120 information lists. This data is only made available for approved studies serving the public interest [24].

### Patient population

**Fig 1:**
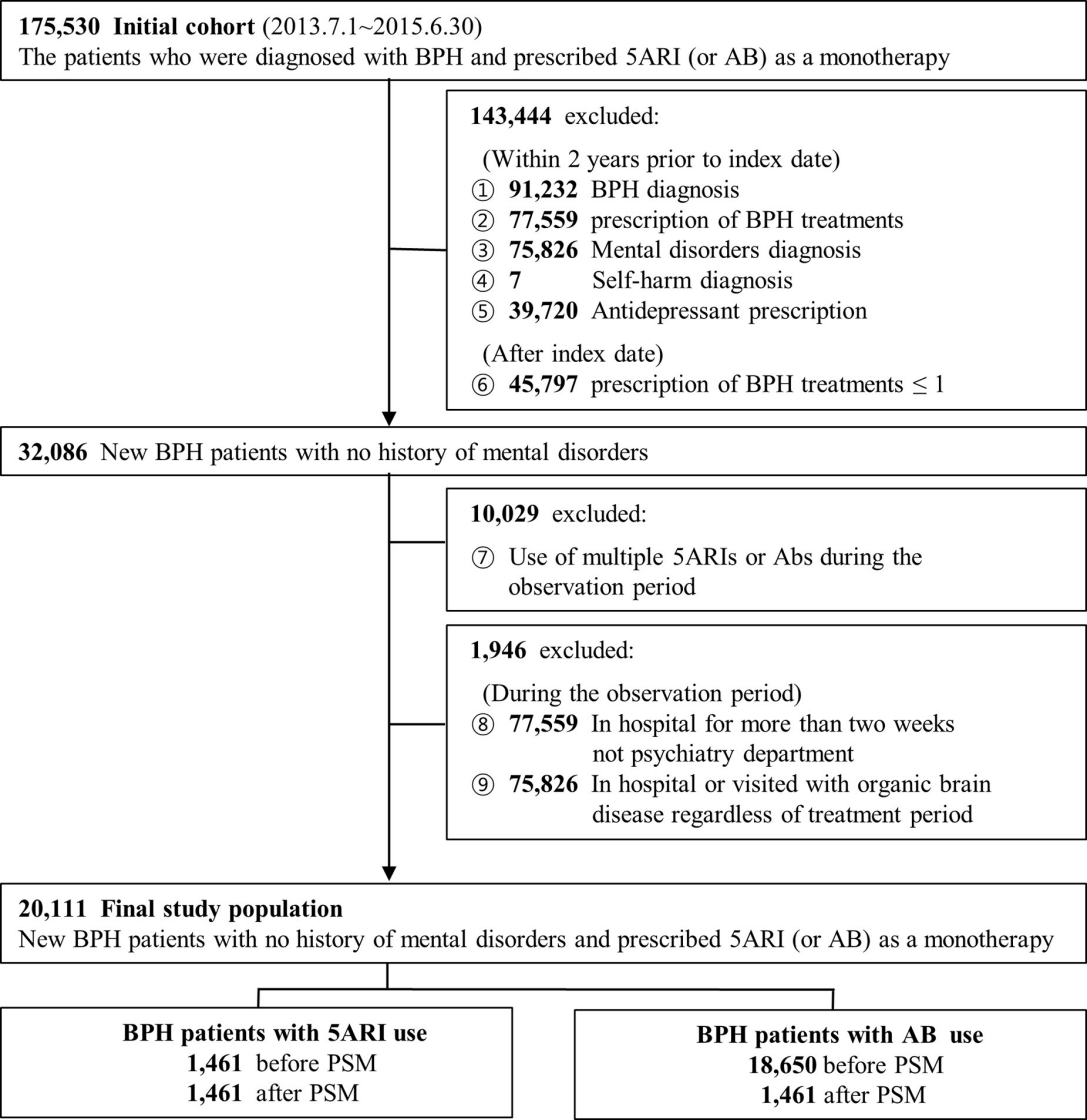
The flow chart of study population (BPH patients with prescription 5ARI or AB). Abbreviations: BPH, benign prostatic hyperplasia; 5ARI, 5alpha reductase inhibitor; AB, alpha blocker; PSM, propensity score matching.

The patients selected for this study were newly diagnosed in men with BPH (ICD-10: N40) and initiated treatments that included either a monotherapy 5ARI or an AB between July 1, 2013, and June 30, 2015. The period was set in consideration of the look-back period and the follow-up period using the latest data available from HIRA (with restrictions on the size of the data that could be provided). We identified an index date of each patient’s first prescription with 5ARI (dutasteride, finasteride) or AB (alfuzosin, doxazosin, silodosin, tamsulosin, terazosin) and classified the patients into two groups (5ARI exposed group or AB control group), and excluded subjects prescribed non-reimbursed drugs (e.g., tadalafil). Within both groups, we excluded patients diagnosed with BPH, mental disorders (ICD-10: F00-F79, F99), and self-harm (ICD-10: X60-X84) and those who were prescribed related medications in the two years before their index date. We also excluded patients who (after the index date) were prescribed BPH treatment less than once, used multiple types of 5ARI or AB, in the hospital for more than two weeks, treated by the psychiatry department, or hospitalized or treated for organic brain disease regardless of the treatment period (to limit the potential confounding of serious concurrent medical illness) [**Fig 1**].

### Outcomes and exposure

The outcomes of the study were defined as mood symptoms associated with 5ARI or AB prescription and classified according to the combination of depression diagnosis and antidepressants as follows: (1) depression (defined as ICD-10 code: F32-F34, F38, F412, F432 and related antidepressants), (2) antidepressants only (defined as be prescribed more than two times), (3) depression diagnosis, (4) anxiety disorder (defined as ICD-10 code: F40-F41), (5) extensive depression (defined as a diagnosis of depression, insomnia or anxiety disorder at least once). It is possible that the study included new diagnosis of depression that occurred shortly after the initial prescription of the BPH treatment. The observation period was from the index date to the occurrence of the outcomes, death, or end of the study (June 30, 2017).

We obtained the prescription information between cohort entry and index dates for all BPH patients and defined the exposure to 5ARI or AB as at least once as a monotherapy. The exposure period was considered to involve the continuous use of 5ARI or AB from the index date. A permissible gap of continuous usage was within 1.5 × the mean of the prescription duration. The end date of treatments was marked as the last prescription date that could be considered continuous + the prescription duration (if the end date of treatment > the end date of study then we considered the latter). There were 12 months of additional observation period after discontinuation of 5ARI/AB to track related symptoms [17].

### Covariates

Based on the index date, we described the demographic information (age) and healthcare use pattern (such as medical insurance type, medical institution type, emergency medical services, hospitalization experience, number of out-patient visits) of patients with BPH. Medical history (such as comorbidities) was also observed for myocardial infarction, congestive heart failure, cardiac arrhythmia, valvular heart disease, hypertension, chronic obstructive pulmonary disease, disturbance of pulmonary circulation, kidney disease, peripheral blood vessel disease, cerebrovascular diseases, diabetes mellitus, liver disease, peptic ulcer, rheumatoid arthritis, neurological disorder, paralysis, malignant tumor, hypothyroidism, coagulopathy, obesity, weight loss, fluid and electrolyte disease, anemia, alcohol abuse, drug abuse, psychosis, and coronary artery disease that had been diagnosed in the year before the index date. Then we also investigated the medications that the BPH patients were taking, including hypotensive agents, hypoglycemic agents, anti-dyslipidemia, nonsteroidal anti-inflammatory drugs (NSAID), narcotic analgesics, hypnotics and sedatives, tranquilizer, steroid, antipsychotic, antimigraine preparations, cardiac glycosides. All patients who had received these drugs in the year before the index date were considered to be taking those medications.

### Statistical analysis

To compare the differences of baseline characteristics between 5ARI and AB users, we examined the frequencies and percentages for the categorical variables and then used the chi-square test, and in some cases, Fisher’s exact test. Next, we calculated the means and standard deviations (SD) for the continuous variables. Then we used propensity score (PS) matching (greedy 1:1 matching) to reduce the potential selection bias in an observational study and balance the distribution between the two groups excluding the confounding variables. A logistic regression model was fitted to estimate the propensity score (i.e., probability of inclusion in the treatment group) [25–27], and standardized difference (STD) was the statistic used for the assessment of covariate balance after PS matching. An STD greater than 0.1 can be considered as a sign of a meaningful imbalance between the study groups. We then used the Cox proportional-hazard regression and estimated the hazard ratios (HR) with 95% confidence intervals (CI) to assess new-onset depression associated with 5ARI use. In addition, we compared the differences of HR according to the before and after PS matching. As a sensitivity analysis, we classified the 5ARI components into finasteride and dutasteride and analyzed the risk of depression in each component of 5ARI compared to the AB group. And then, as a subgroup analysis, we compared the risk of depression in the 5ARI group according to the use of medical institutions based on the index date, that is, hospital and clinic visits. The statistical software for the analysis was SAS Enterprise Guide 6.1 (SAS Institute Inc., Cary, NC) provided by the HIRA via a remote server (https://ras.hira.or.kr/) and statistical detection was tested at an alpha level less than 0.05.

## Results

Among the 1,854,302 men diagnosed with BPH from July 1, 2013, to June 30, 2015, after exclusion criteria, there were 1,461 remaining users of 5ARI (7.3%), and 18,650 (92.7%) AB users. As a result of the one-to-one PS matching, we found that the 1461 patients balanced the covariates between 5ARI and AB users. Baseline characteristics of BPH patients taking 5ARI or AB are listed in Table 1. There were signs of a meaningful imbalance between the two groups before PS matching the medical institution type, admission route, emergency medical services, and malignant tumor (STD greater than 0.1). However, a similar distribution was found between the 5ARI and AB groups in the PS-matched cohort [**Table 1**].

**Table 1:** Baseline characteristics of study population before and after PS matching in 2013.7–2015.6.

Characteristics	Total (N = 20,111)	Before PS matching			After PS matching^a^		
		5ARI (N = 1,461)	AB (N = 18,650)	STD^b^	5ARI (N = 1,461)	AB	STD^b^
						(N = 1,461)	
	No. (%)	No. (%)	No. (%)		No. (%)	No. (%)	
**Age (years)**							
Mean (±SD)	58.2 (±10.7)	57.5 (±10.4)	58.3 (±10.7)	0.0595	57.5 (±10.4)	57.0 (±10.7)	0.0115
<50	4,048 (20.1)	327 (22.4)	3,721 (18.5)		327 (22.4)	334 (22.9)	
50–59	7,116 (35.4)	540 (37.0)	6,576 (32.7)		540 (37.0)	533 (36.5)	
60–69	5,897 (29.3)	395 (27.0)	5,502 (27.4)		395 (27.0)	400 (27.4)	
70+	3,050 (15.2)	199 (13.6)	2,851 (14.2)		199 (13.6)	194 (13.3)	
**Medical insurance type**							
Health insurance	19,661 (97.9)	1,436 (98.3)	18,255 (97.9)	0.0297	1,436 (98.3)	1,427 (97.7)	0.0438
Medical aid/free medical and veterans healthcare	420 (2.1)	25 (1.7)	395 (2.1)		25 (1.7)	34 (2.3)	
**Medical institution type**							
Hospital	9,931 (49.4)	565 (38.7)	9,366 (50.2)	0.2340	565 (38.7)	549 (37.6)	0.0225
Clinic	10,180 (50.6)	896 (61.3)	9,284 (49.8)		896 (61.3)	912 (62.4)	
**Admission route**							
Inpatients	961 (4.8)	16 (1.1)	945 (5.1)	0.2314	16 (1.1)	14 (1.0)	0.0136
Outpatients	19,150 (95.2)	1,445 (98.9)	17,705 (94.9)		1,445 (98.9)	1,447 (99.0)	
**Region**							
Urban(Metropolitan city)	11,796 (58.7)	865 (59.2)	10,931 (58.6)	0.0121	865 (59.2)	841 (57.6)	0.0333
Rural(Province)	8,315 (41.3)	596 (40.8)	7,719 (41.4)		596 (40.8)	620 (42.4)	
**Smoking status**							
Yes	15 (0.1)	≤5 (-)	13 (0.1)	0.0297	≤5 (-)	≤5 (-)	.
No	20,096 (99.9)	1,459 (99.9)	18,637 (99.9)		1,459 (99.9)	1,461 (100.0)	
**Emergency medical services**							
Yes	1,907 (9.5)	88 (6.0)	1,819 (9.8)	0.1387	88 (6.0)	125 (8.6)	0.0975
No	18,204 (90.5)	1,373 (94.0)	16,831 (90.2)		1,373 (94.0)	1,336 (91.4)	
**Hospitalization experience**							
Yes	3,167 (15.7)	200 (13.7)	2,967 (15.9)	0.0625	200 (13.7)	200 (13.7)	0.0000
No	16,944 (84.3)	1,261 (86.3)	15,683 (84.1)		1,261 (86.3)	1,261 (86.3)	
**Number of outpatient visit**							
0–9	5,753 (28.6)	473 (32.4)	5,280 (28.3)	0.0885	473 (32.4)	445 (30.5)	0.0413
10–19	6,890 (34.3)	488 (33.4)	6,402 (34.3)		488 (33.4)	493 (33.7)	
20–29	3,934 (19.6)	276 (18.9)	3,658 (19.6)		276 (18.9)	298 (20.4)	
≥30	3,534 (17.6)	224 (15.3)	3,310 (17.7)		224 (15.3)	225 (15.4)	
**Concomitants**							
Hypotensive agents	3,059 (39.0)	218 (39.7)	2,841 (38.9)	0.0087	218 (39.7)	559 (38.3)	0.0096
Hypoglycemic agents	7,834 (15.2)	580 (14.9)	7,254 (15.2)	0.0164	580 (14.9)	223 (15.3)	0.0295
Anti-dyslipidemia	4,866 (24.2)	381 (26.1)	4,485 (24.0)	0.0468	381 (26.1)	343 (23.5)	0.0603
NSAIDs	7,390 (36.7)	492 (33.7)	6,898 (37.0)	0.0693	492 (33.7)	524 (35.9)	0.0460
Narcotic analgesics	888 (4.4)	55 (3.8)	833 (4.5)	0.0353	55 (3.8)	48 (3.3)	0.0260
Hypnotics and sedatives tranquilizer	2,081 (10.3)	127 (8.7)	1,954 (10.5)	0.0606	127 (8.7)	142 (9.7)	0.0355
Steroid	7,334 (36.5)	523 (35.8)	6,811 (36.5)	0.0150	523 (35.8)	547 (37.4)	0.0341
Antipsychotic	12 (0.1)	≤5 (-)	12 (0.1)	.	≤5 (-)	≤5 (-)	.
Antimigraine preparations	161 (0.8)	11 (0.8)	150 (0.8)	0.0058	11 (0.8)	10 (0.7)	0.0081
Cardiac glycosides	85 (0.4)	≤5 (-)	81 (0.4)	0.0270	≤5 (-)	≤5 (-)	0.0497
Interferons	≤5 (-)	≤5 (-)	≤5 (-)	.	≤5 (-)	≤5 (-)	.
**Charlson comorbidity index (CCI)**							
Mean (±SD)	1.5 (±1.6)	1.4 (±1.6)	1.5 (±1.6)	0.0036	1.4 (±1.6)	1.3 (±1.6)	0.0139
0	7,003 (34.8)	545 (37.3)	6,458 (34.6)		545 (37.3)	580 (39.7)	
1	5,384 (26.8)	389 (26.6)	4,995 (26.8)		389 (26.6)	398 (27.2)	
2	3,565 (17.7)	248 (17.0)	3,317 (17.8)		248 (17.0)	207 (14.2)	
≥3	4,159 (20.7)	279 (19.1)	3,880 (20.8)		279 (19.1)	276 (18.9)	
**Comorbidities**							
Myocardial infarction	263 (1.3)	16 (1.1)	247 (1.3)	0.0210	16 (1.1)	6 (0.4)	0.0792
Congestive heart failure	496 (2.5)	44 (3)	449 (2.4)	0.0372	44 (3)	39 (2.7)	0.0206
Cardiac arrhythmia	731 (3.6)	46 (3.1)	685 (3.7)	0.0289	46 (3.1)	53 (3.6)	0.0265
Valvular heart disease	84 (0.4)	5 (0.3)	79 (0.4)	0.0132	5 (0.3)	5 (0.3)	0.0000
Hypertension	7,057 (35.1)	534 (36.6)	6,523 (35.0)	0.0329	534 (36.6)	516 (35.3)	0.0257
Chronic obstructive pulmonary disease	4,771 (23.7)	322 (22)	4,449 (23.9)	0.0432	322 (22)	322 (22)	0.0000
Disturbance of pulmonary circulation	17 (0.1)	≤5 (-)	14 (0.1)	0.0348	≤5 (-)	≤5 (-)	0.0140
Kidney disease	392 (1.9)	20 (1.4)	372 (2)	0.0487	20 (1.4)	21 (1.4)	0.0058
Peripheral blood vessel disease	1,738 (8.6)	130 (8.9)	1,608 (8.6)	0.0098	130 (8.9)	111 (7.6)	0.0473
Cerebrovascular diseases	584 (2.9)	36 (2.5)	548 (2.9)	0.0293	36 (2.5)	23 (1.6)	0.0633
Dementia	29 (0.1)	≤5 (-)	29 (0.2)	.	≤5 (-)	≤5 (-)	.
Diabetes mellitus	4,851 (24.1)	338 (23.1)	4,513 (24.2)	0.0250	338 (23.1)	340 (23.3)	0.0032
Liver disease	4,992 (24.8)	362 (24.8)	4,630 (24.8)	0.0011	362 (24.8)	356 (24.4)	0.0095
Peptic ulcer	4,147 (20.6)	299 (20.5)	3,848 (20.6)	0.0041	299 (20.5)	268 (18.3)	0.0537
Rheumatoid arthritis	485 (2.4)	39 (2.7)	446 (2.4)	0.0177	39 (2.7)	38 (2.6)	0.0043
Neurological disorder	124 (0.6)	8 (0.5)	116 (0.6)	0.0098	8 (0.5)	10 (0.7)	0.0175
Paralysis	18 (0.1)	≤5 (-)	16 (0.1)	0.0153	≤5 (-)	≤5 (-)	0.0214
Malignant tumor	1,684 (8.4)	86 (5.9)	1,598 (8.6)	0.1037	86 (5.9)	86 (5.9)	0.0000
AIDS/HIV	6 (0.0)	≤5 (-)	6 (0.0)	.	≤5 (-)	≤5 (-)	.
Hypothyroidism	539 (2.7)	31 (2.1)	508 (2.7)	0.0392	31 (2.1)	45 (3.1)	0.0602
Coagulopathy	253 (1.3)	10 (0.7)	243 (1.3)	0.0624	10 (0.7)	5 (0.3)	0.0479
Obesity	10 (0.0)	≤5 (-)	9 (0.0)	.	≤5 (-)	0 (0)	.
Weight loss	142 (0.7)	5 (0.3)	137 (0.7)	0.0536	5 (0.3)	7 (0.5)	0.0214
Fluid and electrolyte disease	395 (2.0)	29 (2.0)	366 (2.0)	0.0016	29 (2.0)	18 (1.2)	0.0599
Anemia	696 (3.5)	45 (3.1)	651 (3.5)	0.0230	45 (3.1)	53 (3.6)	0.0304
Alcohol abuse/Drug abuse	482 (2.4)	28 (1.9)	454 (2.4)	0.0355	28 (1.9)	23 (1.6)	0.0261
Psychosis	≤5 (-)	≤5 (-)	≤5 (-)	.	≤5 (-)	≤5 (-)	.
Coronary artery disease	288 (1.4)	19 (1.3)	269 (1.4)	0.0122	19 (1.3)	7 (0.5)	0.0875

^a^ The BPH patients with a 5ARI prescription were 1:1 matched to patients prescribed an AB. The propensity score was estimated using Logistic regression model adjusted for age, and comorbidities, concomitant drugs in the 1 year prior to the index date.

^b^ The standardized difference (STD) was used for the assessment of covariate balance after PS matching. A STD greater than 0.1 can be considered as a sign meaningful imbalance between study groups.

^c^ The health care uilizations (such as medical insurance type, medical institution type, admission route, Region and so on) were defined on the index date.

^d^ Due to privacy issues, the values ≤5 are suppressed.

**Abbreviation**: *PS*, propensity score; *5ARI*, 5alpha reductase inhibitor; *AB*, alpha blocker; *STD*, standardized difference; *SD*, standard deviation; *NSAIDs*, Non-steroidal anti-inflammatory drugs; *AIDS/HIV*, acquired immune deficiency syndrome/human immunodeficiency virus.

### Risk of depression associated with 5ARI use

**Fig 2:**
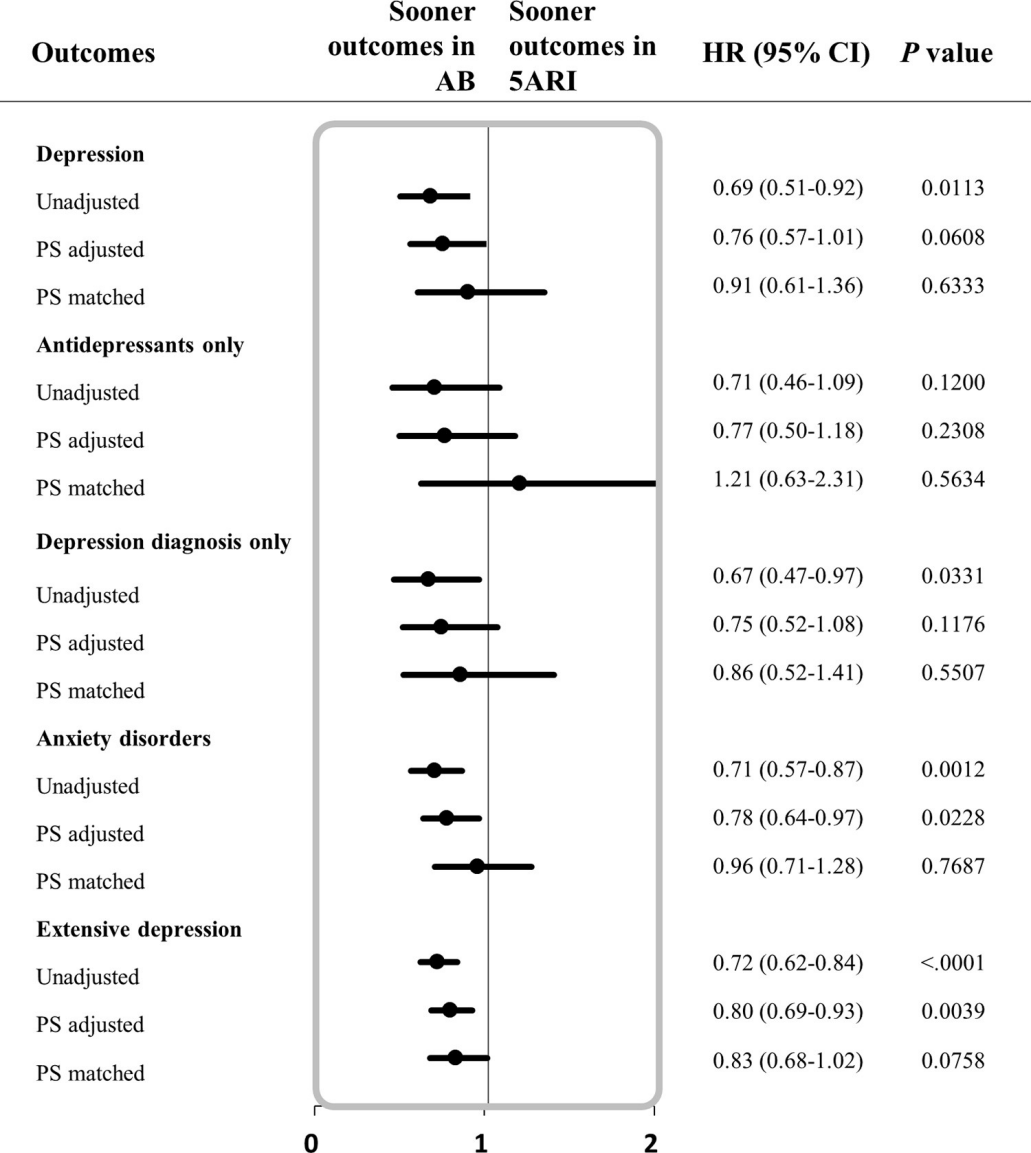
Forest plot for hazard ratio related to depressive symptoms among BPH patients exposed to 5ARI versus AB. The study outcomes: depression (classified into 3 categories based on the combination of diagnosis and antidepressants), anxiety disorder, and extensive depression. Abbreviations: BPH, Benign prostatic hyperplasia; 5ARI, 5alpha reductase inhibitor; AB, alpha blocker; HR, hazard ratio; CI, confidence intervals; PS adjusted, adjusted by propensity score quintiles; PS matched, matched 1:1 by propensity score.

The analysis of the risk of developing depression, anxiety disorder and extensive depression associated with the use of 5ARI compared to AB in BPH patients is as follows. First, when the patients with BPH were diagnosed with depression and prescribed antidepressants, the risk of depression in the 5ARI-treated group was lower than that in the AB-treated group (HR: 0.69; 95% CI [0.51–0.92]), but not after the PS matching (HR: 0.91; 95% CI [0.61–1.36]). Second, When the depression was defined as two or more prescription antidepressants, the risk of depression in the 5ARI group was not different (before and after PS matching; HR: 0.71, 95% CI [0.46–1.09] and HR: 1.21, 95% CI [0.63–2.31]) regardless of PS matching. Third, when patients had been diagnosed with depression more than once on a diagnostic code (ICD-10), the risk of depression in the 5ARI users was statistically lower than the AB users before PS matching (HR: 0.67; 95% CI [0.47–0.97]), but the difference was not noticeable after PS matching (HR: 0.86; 95% CI [0.52–1.41)]. In addition, there were no detectable differences in the risk of anxiety disorder and extensive depression due to the use of 5ARI versus the control group [**Table 2**] [**Fig 2**].

**Table 2:** Risk of depression and anxiety disorders among BPH patients exposed to 5ARI versus AB.

Outcomes^a^	Total (N = 20,111)	5ARI (N = 1,461)		AB (N = 18,650)		HR (95% CI)	*P* value^b^
	No. of events (%)	N	No. of events (%)	N	No. of events (%)		
**Depression**							
Unadjusted	866 (4.3)	1,461	48 (3.3)	18,650	1,413 (7.6)	0.69 (0.51–0.92)	0.0113
PS adjusted^c^	866 (4.3)	1,461	48 (3.3)	18,650	1,413 (7.6)	0.76 (0.57–1.01)	0.0608
PS matched^d^	96 (3.3)	1,461	48 (3.3)	1,461	48 (3.3)	0.91 (0.61–1.36)	0.6333
**Antidepressants only**							
Unadjusted	374 (1.9)	1,461	22 (1.5)	18,650	1,439 (7.7)	0.71 (0.46–1.09)	0.1200
PS adjusted	374 (1.9)	1,461	22 (1.5)	18,650	1,439 (7.7)	0.77 (0.50–1.18)	0.2308
PS matched	38 (1.3)	1,461	22 (1.5)	1,461	16 (1.1)	1.21 (0.63–2.31)	0.5634
**Depression diagnosis only**							
Unadjusted	567 (2.8)	1,461	31 (2.1)	18,650	1,430 (7.7)	0.67 (0.47–0.97)	0.0331
PS adjusted	567 (2.8)	1,461	31 (2.1)	18,650	1,430 (7.7)	0.75 (0.52–1.08)	0.1176
PS matched	63 (2.2)	1,461	31 (2.1)	1,461	32 (2.2)	0.86 (0.52–1.41)	0.5507
**Anxiety disorders**							
Unadjusted	1,634 (8.1)	1,461	93 (6.4)	18,650	1,368 (7.3)	0.71 (0.57–0.87)	0.0012
PS adjusted	1,634 (8.1)	1,461	93 (6.4)	18,650	1,368 (7.3)	0.78 (0.64–0.97)	0.0228
PS matched	180 (6.2)	1,461	93 (6.4)	1,461	87 (6.0)	0.96 (0.71–1.28)	0.7687
**Extensive depression**							
Unadjusted	3,125 (15.5)	1,461	183 (12.5)	18,650	1,278 (6.9)	0.72 (0.62–0.84)	< .0001
PS adjusted	3,125 (15.5)	1,461	183 (12.5)	18,650	1,278 (6.9)	0.80 (0.69–0.93)	0.0039
PS matched	379 (13.0)	1,461	183 (12.5)	1461	196 (13.4)	0.83 (0.68–1.02)	0.0758

^**a**^ The study outcomes were defined as follows: depression (classified into 3 categories based on the combination of diagnosis and antidepressants), anxiety disorder, and extensive depression.

^**b**^ *P* values ≤ 0.05 was considered statistically significant.

^**c**^ Adjusted by propensity score quintiles.

^**d**^ Propensity score matching was used to compare 1:1 within 0.1 caliper without replacement.

**Abbreviation**: *5ARI*, 5alpha reductase inhibitor; *AB*, alpha blocker; *HR*, hazard ratio; *CI*, confidence intervals; *PS*, propensity score.

### Sensitivity analysis

**Fig 3:**
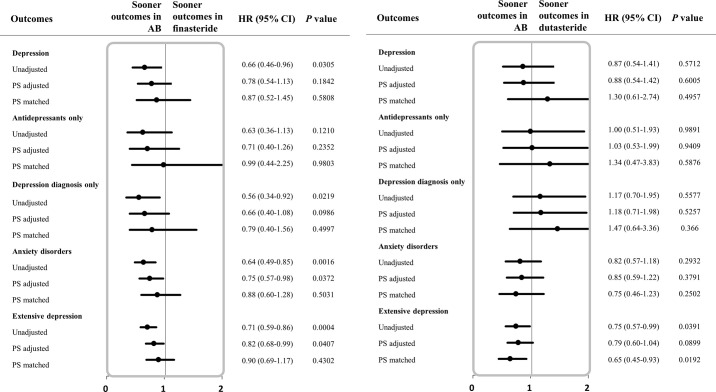
a. Forest plot for hazard ratio related to depressive symptoms among BPH patients exposed to finasteride versus AB. b. Forest plot for hazard ratio related to depressive symptoms among BPH patients exposed to dutasteride versus AB. The study outcomes: depression (classified into 3 categories based on the combination of diagnosis and antidepressants), anxiety disorder, and extensive depression. Abbreviations: BPH, Benign prostatic hyperplasia; AB, alpha blocker; HR, hazard ratio; CI, confidence intervals; PS adjusted, adjusted by propensity score quintiles; PS matched, matched 1:1 by propensity score.

**Depression risk associated with finasteride or dutasteride.** We classified 5ARI by the ingredients (finasteride and dutasteride) of medications and analyzed the risk of depression by comparison with the AB users. A total of 961 patients in the finasteride group and 363 patients in the dutasteride group were included in the subgroup analysis; 137 patients who were prescribed received both finasteride and dutasteride during the follow-up period were excluded from this analysis. The finasteride-treated group had a lower risk of depression (HR: 0.66; 95% CI [0.46–0.96]), anxiety disorder (HR: 0.64; 95% CI [0.49–0.85]), and extensive depression (HR: 0.71; 95% CI [0.59–0.86]) before PS matching compared to the AB-treated group. However, after PS matching, there was no difference in risk of depression between the two groups. Similarly, there was no detectable difference between the dutasteride groups and AB groups, but only the dutasteride-dosing group had lower risk than the AB-dosing group for extensive depression (before and after PS matching; HR: 0.75; 95% CI [0.57–0.99] and HR: 0.65; 95% CI [0.45–0.93]) [**Table 3**] [**Fig 3A and 3B**].

**Table 3:** Risk of depression anxiety disorders and extensive depression outcomes stratified by initial 5ARI type.

Outcomes^a^	Total (N = 19,611)	Finasteride (N = 961)		AB (N = 18,650)		HR (95% CI)	*P* value^b^
	No. of events (%)	N	No. of events (%)	N	No. of events (%)		
**Finasteride**							
**Depression**							
Unadjusted	847 (4.3)	961	29 (3.0)	18,650	818 (4.4)	0.66 (0.46–0.96)	0.0305
PS adjusted^c^	847 (4.3)	961	29 (3.0)	18,650	818 (4.4)	0.78 (0.54–1.13)	0.1842
PS matched^d^	59 (0.3)	961	29 (3.0)	961	30 (0.2)	0.87 (0.52–1.45)	0.5808
**Antidepressants only**							
Unadjusted	364 (1.9)	961	12 (1.2)	18,650	352 (1.9)	0.63 (0.36–1.13)	0.1210
PS adjusted	364 (1.9)	961	12 (1.2)	18,650	352 (1.9)	0.71 (0.40–1.26)	0.2352
PS matched	23 (0.1)	961	12 (1.2)	961	11 (0.1)	0.99 (0.44–2.25)	0.9803
**Depression diagnosis only**							
Unadjusted	552 (2.8)	961	16 (1.7)	18,650	536 (2.9)	0.56 (0.34–0.92)	0.0219
PS adjusted	552 (2.8)	961	16 (1.7)	8,650	536 (2.9)	0.66 (0.40–1.08)	0.0986
PS matched	34 (0.2)	961	16 (1.7)	961	18 (0.1)	0.79 (0.40–1.56)	0.4997
**Anxiety disorders**							
Unadjusted	1,594 (8.1)	961	53 (5.5)	18,650	1,541 (8.3)	0.64 (0.49–0.85)	0.0016
PS adjusted	1,594 (8.1)	961	53 (5.5)	18,650	1,541 (8.3)	0.75 (0.57–0.98)	0.0372
PS matched	108 (0.6)	961	53 (5.5)	961	55 (0.3)	0.88 (0.60–1.28)	0.5031
**Extensive depression**							
Unadjusted	3,055 (15.6)	961	113 (11.8)	18,650	2,942 (15.8)	0.71 (0.59–0.86)	0.0004
PS adjusted	3,055 (15.6)	961	113 (11.8)	8,650	2,942 (15.8)	0.82 (0.68–0.99)	0.0407
PS matched	227 (1.2)	961	113 (11.8)	961	114 (0.6)	0.90 (0.69–1.17)	0.4302
**Dutasteride**							
**Depression**							
Unadjusted	835 (4.4)	363	17 (4.7)	18,650	818 (4.4)	0.87 (0.54–1.41)	0.5712
PS adjusted^c^	835 (4.4)	363	17 (4.7)	18,650	818 (4.4)	0.88 (0.54–1.42)	0.6005
PS matched^d^	29 (0.2)	363	17 (4.7)	363	12 (0.1)	1.30 (0.61–2.74)	0.4957
**Antidepressants only**							
Unadjusted	361 (1.9)	363	9 (2.5)	18,650	352 (1.9)	1.00 (0.51–1.93)	0.9891
PS adjusted	361 (1.9)	363	9 (2.5)	18,650	352 (1.9)	1.03 (0.53–1.99)	0.9409
PS matched	15 (0.1)	363	9 (2.5)	363	6 (0.0)	1.34 (0.47–3.83)	0.5876
**Depression diagnosis only**							
Unadjusted	551 (2.9)	363	15 (4.1)	18,650	536 (2.9)	1.17 (0.70–1.95)	0.5577
PS adjusted	551 (2.9)	363	15 (4.1)	8,650	536 (2.9)	1.18 (0.71–1.98)	0.5257
PS matched	24 (0.1)	363	15 (4.1)	363	9 (0.0)	1.47 (0.64–3.36)	0.366
**Anxiety disorders**							
Unadjusted	1,571 (8.3)	363	30 (8.3)	18,650	1541 (8.3)	0.82 (0.57–1.18)	0.2932
PS adjusted	1,571 (8.3)	363	30 (8.3)	18,650	1,541 (8.3)	0.85 (0.59–1.22)	0.3791
PS matched	64 (0.3)	363	30 (8.3)	363	34 (0.2)	0.75 (0.46–1.23)	0.2502
**Extensive depression**							
Unadjusted	2,994 (15.7)	363	52 (14.3)	18,650	2,942 (15.8)	0.75 (0.57–0.99)	0.0391
PS adjusted	2,994 (15.7)	363	52 (14.3)	8,650	2,942 (15.8)	0.79 (0.60–1.04)	0.0899
PS matched	120 (0.6)	363	52 (14.3)	363	68 (0.4)	0.65 (0.45–0.93)	0.0192

^**a**^ The study outcomes were defined as follows: depression (classified into 3 categories based on the combination of diagnosis and antidepressants), anxiety disorder, and extensive depression.

^**b**^ *P* values ≤ 0.05 was considered statistically significant.

^**c**^ Adjusted by propensity score quintiles.

^**d**^ Propensity score matching was used to compare 1:1 within 0.1 caliper without replacement.

**Abbreviation**: *5ARI*, 5alpha reductase inhibitor; *AB*, alpha blocker; *HR*, hazard ratio; *CI*, confidence intervals; *PS*, propensity score.

### Subgroup analysis

**Fig 4:**
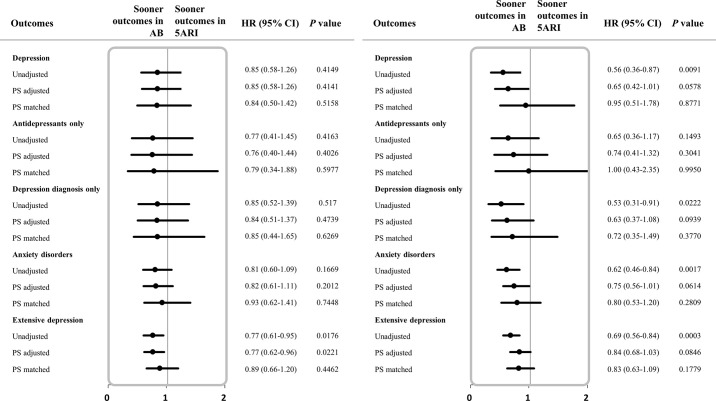
a. Forest plot for hazard ratio related to depressive symptoms among BPH patients exposed to 5ARI versus AB in hospital. b. Forest plot for hazard ratio related to depressive symptoms among BPH patients exposed to 5ARI versus AB in clinic. The study outcomes: depression (classified into 3 categories based on the combination of diagnosis and antidepressants), anxiety disorder, and extensive depression. Abbreviations: BPH, Benign prostatic hyperplasia; 5ARI, 5alpha reductase inhibitor; AB, alpha blocker; HR, hazard ratio; CI, confidence intervals; PS adjusted, adjusted by propensity score quintiles; PS matched, matched 1:1 by propensity score.

**Depression risk associated with 5ARI in BPH patients visiting the hospital on the index date.** The risk of 5ARI-related depression was compared with the AB group according to the visit patterns of a medical institution (hospital and clinic) on the index date. There was no statistical difference in the risk of depression between the 565 patients taking 5ARI and the 9,366 patients taking AB who visited the hospital. Among the 10,180 patients who visited the clinic, the 5ARI-treated group (896 patients) had a lower risk of depression, anxiety disorder, and extensive depression compared to the AB-treated group (9,284 patients) before PS matching but did not show any relevant results after PS matching [**Table 4**] [**Fig 4A and 4B**].

**Table 4:** Risk of depression anxiety disorders and extensive depression outcomes among BPH patients stratified by medical institution type at index date of cohort.

Outcomes^a^	Total (N = 19,297)	Hospital (N = 9,931)		AB (N = 9,366)		HR (95% CI)	*P* value^b^
	No. of events (%)	N	No. of events (%)	N	No. of events (%)		
**Hospital**							
**Depression**							
Unadjusted	482 (4.7)	565	27 (3)	9,366	455 (4.9)	0.85 (0.58–1.26)	0.4149
PS adjusted^c^	482 (4.7)	565	27 (3)	9,366	455(4.9)	0.85 (0.58–1.26)	0.4141
PS matched^d^	56 (0.6)	565	27 (3)	565	29 (0.3)	0.84 (0.50–1.42)	0.5158
**Antidepressants only**							
Unadjusted	187 (1.8)	565	10 (1.1)	9,366	177 (1.9)	0.77 (0.41–1.45)	0.4163
PS adjusted	187 (1.8)	565	10 (1.1)	9,366	177 (1.9)	0.76 (0.40–1.44)	0.4026
PS matched	21 (0.2)	565	10 (1.1)	565	11 (0.1)	0.79 (0.34–1.88)	0.5977
**Depression diagnosis only**							
Unadjusted	300 (2.9)	565	17 (1.9)	9,366	283 (3)	0.85 (0.52–1.39)	0.5170
PS adjusted	300 (2.9)	565	17 (1.9)	9366	283 (3)	0.84 (0.51–1.37)	0.4739
PS matched	35 (0.3)	565	17 (1.9)	565	18 (0.2)	0.85 (0.44–1.65)	0.6269
**Anxiety disorders**							
Unadjusted	862 (8.5)	565	46 (5.1)	9,366	816 (8.8)	0.81 (0.60–1.09)	0.1669
PS adjusted	862 (8.5)	565	46 (5.1)	9,366	816 (8.8)	0.82 (0.61–1.11)	0.2012
PS matched	90 (0.9)	565	46 (5.1)	565	44 (0.5)	0.93 (0.62–1.41)	0.7448
**Extensive depression**							
Unadjusted	1,636 (16.1)	565	83 (9.3)	9,366	1,553 (16.7)	0.77 (0.61–0.95)	0.0176
PS adjusted	1,636 (16.1)	565	83 (9.3)	9,366	1,553 (16.7)	0.77 (0.62–0.96)	0.0221
PS matched	167 (1.6)	565	83 (9.3)	565	84 (0.9)	0.89 (0.66–1.20)	0.4462
**Clinic**							
**Depression**							
Unadjusted	384 (3.8)	896	21 (2.3)	9,284	363 (3.9)	0.56 (0.36–0.87)	0.0091
PS adjusted^c^	384 (3.8)	896	21 (2.3)	9,284	363 (3.9)	0.65 (0.42–1.01)	0.0578
PS matched^d^	40 (0.4)	896	21 (2.3)	896	19 (0.2)	0.95 (0.51–1.78)	0.8771
**Antidepressants only**							
Unadjusted	187 (1.8)	896	12 (1.3)	9,284	175 (1.9)	0.65 (0.36–1.17)	0.1493
PS adjusted	187 (1.8)	896	12 (1.3)	9,284	175 (1.9)	0.74 (0.41–1.32)	0.3041
PS matched	22 (0.2)	896	12 (1.3)	896	10 (0.1)	1.00 (0.43–2.35)	0.9950
**Depression diagnosis only**							
Unadjusted	267 (2.6)	896	14 (1.6)	9,284	253 (2.7)	0.53 (0.31–0.91)	0.0222
PS adjusted	267 (2.6)	896	14 (1.6)	9,284	253 (2.7)	0.63 (0.37–1.08)	0.0939
PS matched	30 (0.3)	896	14 (1.6)	896	16 (0.2)	0.72 (0.35–1.49)	0.3770
**Anxiety disorders**							
Unadjusted	772 (7.6)	896	47 (5.2)	9,284	725 (7.8)	0.62 (0.46–0.84)	0.0017
PS adjusted	772 (7.6)	896	47 (5.2)	9,284	725 (7.8)	0.75 (0.56–1.01)	0.0614
PS matched	96 (0.9)	896	47 (5.2)	896	49 (0.5)	0.80 (0.53–1.20)	0.2809
**Extensive depression**							
Unadjusted	1,489 (14.6)	896	100 (11.2)	9,284	1,389 (15)	0.69 (0.56–0.84)	0.0003
PS adjusted	1,489 (14.6)	896	100 (11.2)	9,284	1,389 (15)	0.84 (0.68–1.03)	0.0846
PS matched	203 (2.0)	896	100 (11.2)	896	103 (1.1)	0.83 (0.63–1.09)	0.1779

^**a**^ The study outcomes were defined as follows: depression (classified into 3 categories based on the combination of diagnosis and antidepressants), anxiety disorder, and extensive depression.

^**b**^ *P* values ≤ 0.05 was considered statistically significant.

^**c**^ Adjusted by propensity score quintiles.

^**d**^ Propensity score matching was used to compare 1:1 within 0.1 caliper without replacement.

**Abbreviation**: *5ARI*, 5alpha reductase inhibitor; *AB*, alpha blocker; *HR*, hazard ratio; *CI*, confidence intervals; *PS*, propensity score.

## Discussion

The present study found no detectable difference in the risk of depression between 5ARI and AB. The risk of depression in the 5ARI-treated group was lower than in the AB-treated group, but not after PS matching. Subgroup analysis indicated that type of 5ARI had no effect on depression risk, considering adjusted HR for finasteride and dutasteride which are not statistical association. Also, regardless of the different patterns of health care utilization, we found no difference in the risk of depression and anxiety disorder between 5ARI and AB users.

Hagberg et al. [21] analyzed 77,732 BPH patients who were prescribed 5ARI or AB using the UK Clinical Practice Research Datalink (1992–2013). As a result of comparing 2,842 patients with depression and 11,333 people without depression, the study reported that the incidence rate of depression was 7.6 (95% CI [6.9–8.3]) for only 5ARI use, similar for only AB use (incidence rate: 7.8, 95% CI [7.5–8.1]). Also, the risk of depression in the 5ARI-treated group was similar, in that study, to the AB-treated group after adjustment, which is consistent with our results. Results of the prior retrospective cohort study [28] in the USA indicated that there was no difference in the effect of 5ARI, for all causes of mortality, compared to AB among BPH and LUTS patients.

Furthermore, it has been reported that increased risk of depression is associated with a longer duration of BPH regardless of drug exposure [21]. In the cohort analyses using UK General Practice Research Database, the risk of depression in men with BPH was higher compared to those without BPH, but was not different for men exposed to alpha1-blockers (or finasteride) versus those unexposed when adjusted for the presence of BPH [29]. Also, in general, these patients are associated with depression [30, 31]. This indicates the inability to exclude depression due to BPH. Although our study indicated that there is no noticeable difference in the association of drug-induced depression between 5ARI and AB, management of BPH requires close attention. In other words, early detection, and prompt treatment to depression among BPH patients by close observation of mood and depression-related symptoms is essential.

A retrospective cohort study by Welk analyzed 93,197 men (exposed and control groups, respectively) aged 66 years or older who were first prescribed finasteride or dutasteride using the Ontario administrative data source, Canada (2003, 1 year). The risk of self-harm was higher in 5ARI users compared to non-5ARI users during the initial period, but there was detectable no significant difference 1.5 years after study initiation. However, in contrast to our findings, 5ARI treatment increased the risk of depression in the study conducted by Welk et al. A difference in the control group might explain that result. Because Welk selected non-5ARI users as a control group, thus, the proportion of healthy patients might have been higher than in the control group of our study.

In the United States, the Post-Finasteride Syndrome Foundation was established to continuously study, prevent, and manage serious side effects such as suicidal ideation after using finasteride [32]. South Korea has one of the highest suicide rates, although the prevalence of depression has been reported to be low compared to other countries. However, in a recent study, the prevalence of depression using sample data of one million people (2002–2013) increased from 2.8% to 5.3% [33]. As a result of surveying the prevalence of depression in OECD countries in 2020, Korea showed the highest. Although the risk of depression related to 5ARI use for the treatment of BPH did not increase in this study, health professionals must prudentially prescribe medication, and be aware that safety issues related to finasteride are routinely raised. Also, it is necessary to monitor the incidence of depression after drug use more carefully than in other countries.

A strength of this study is that it represents a large population from the South Korean national insurance claims data. Furthermore, we tried to minimize the effect of confounding factors other than exposure to 5ARI by applying statistical propensity scores matching. However, it is difficult to completely exclude the effects of clinical and social confounding factors, which are not measured in the observational study. The inaccuracy of coding and incompleteness of records might be possible due to the nature of claims data. Also, patients using 5ARI for the treatment of alopecia, not BPH, might have been included in this study, inadvertently. In addition, there was a limit on the size of available data according to HIRA’s database usage guidelines, but various large-scale studies need to be conducted starting with this study.

Nevertheless, it is vital that our study provides scientific evidence assessing the risk of depression associated with the use of 5ARI for BPH patients, based on real-world data collected from clinical practices. The study did not compare BPH patients who were not prescribed medication with those who were prescribed 5ARI or AB. This is because BPH patients who are not taking medications and those taking 5ARI may have different BPH severity. According to the Korean clinical guidelines, alpha-blockers (AB) are primarily used to improve urination symptoms in patients with moderate to severe lower urinary tract symptoms, and 5ARI is used to reduce the size of the prostate through long-term use, improve urination symptoms, and reduce the risk of complications such as surgery. Although there has been no large-scale epidemiological study demonstrating the safety of finasteride in South Korea, this study aimed to clarify the safety of 5ARI by conducting an observational study that considered the clinical environment and characteristics of the BPH patients. The results of our study suggest that selecting a therapeutic strategy (such as 5ARI or AB) for BPH based depression risk is unnecessary. The risk of depression cannot be entirely excluded for the entire duration of BPH treatment. Therefore, it is necessary to closely observe the occurrence of depression-related symptoms for patients and related safety issues.

## Conclusions

In South Korea, the risk of depression associated with 5ARI use was not meaningfully different from AB use. However, the previous studies have shown conflicting results, and in the United States, the Post-Finasteride Syndrome Foundation continues to manage the crucial side effects after using finasteride. Therefore, we suggest health-care providers select/consider the appropriate treatment for BPH taking into account both the risk of depression and treatment benefits.
